# The nucleoid-associated protein subunit HupA positively regulates the Pqs system and pyocyanin production in *Pseudomonas aeruginosa*

**DOI:** 10.1128/aem.02425-25

**Published:** 2026-03-09

**Authors:** Zeling Xu, Jiahui Huang, Weiyan Wu, Jiayi Huang, Huiluo Cao, Shuzhen Chen, Yuying Zhao, Tae-Jin Park, Hui Zhou, Li Li

**Affiliations:** 1 Guangdong Province Key Laboratory of Microbial Signals and Disease Control, State Key Laboratory of Green Pesticide, Integrative Microbiology Research Centre, South China Agricultural University 12526, Guangzhou, China; 2 Department of Microbiology, Li Ka Shing Faculty of Medicine, The University of Hong Kong 25809, Hong Kong SAR, China; 3 HME LAB Co., Ltd, Suwon-si, Republic of Korea; 4 Department of Infectious Diseases, The Second Affiliated Hospital, Zhejiang University School of Medicine 89681 , Hangzhou, China; 5 Women and Children's Hospital, Southern University of Science and Technology 90405 , Guangzhou, China; Indiana University Bloomington, Bloomington, Indiana, USA

**Keywords:** *P. aeruginosa*, quorum sensing, pyocyanin, nucleoid-associated protein, genome-wide mutagenesis

## Abstract

*Pseudomonas aeruginosa,* a ubiquitous opportunistic pathogen, coordinates virulence gene expression primarily through three quorum-sensing (QS) systems: Las, Rhl, and Pqs. Despite the functions of QS systems in *P. aeruginosa* being relatively well-documented, the regulatory mechanisms modulating their activities remain largely unexplored. Here, we isolated and characterized a Las-null strain, PA_HN008, which overproduces pyocyanin (PYO) at the late growth stage due to its active RhlI-RhlR-PqsE pathway. Leveraging PYO production as a phenotypic marker, we conducted a genome-wide screening for upstream QS modulators and successfully identified PA5348 (designated as HupA), the α subunit of the nucleoid-associated protein HU, as a specific activator of the Pqs system and PYO production. RNA-seq and protein-DNA interaction assays further revealed that HupA directly binds to the promoters of *pqs* and *phz* genes, thereby inducing PqsE expression and PYO production. This study demonstrates that HupA acts as a novel transcriptional factor to control QS activity and virulence gene expression in *P. aeruginosa*, which not only reveals the regulatory function of the HU subunit HupA on virulence factor production but also uncovers the sequence-specific DNA-binding capability of HupA for stimulating gene transcription.

**IMPORTANCE:**

The nucleoid-associated protein HU is abundant and evolutionarily conserved in bacteria, which typically binds DNA in a sequence-nonspecific manner with high affinity to abnormal DNA structures during DNA damage and then contributes to DNA compaction, replication, recombination, and other physiological functions. However, the potential involvement of HU in regulating quorum sensing (QS), a critical cell-to-cell communication system governing bacterial virulence factor production, is poorly understood. In this study, we demonstrated that the α subunit of the HU protein, namely HupA, is a key regulator that activates the Pqs system and pyocyanin production through genome-wide screening in a clinical isolate PA_HN008. This study reveals the capability of the HU subunit to selectively bind target DNA sequences and stimulate QS activity as well as virulence factor production. Our findings provide novel insights into the biochemical properties of the HU subunit and its regulatory role in bacterial virulence.

## INTRODUCTION

*Pseudomonas aeruginosa* is a ubiquitous gram-negative opportunistic pathogen causing severe acute and chronic infections in immunocompromised individuals, including patients with cystic fibrosis, burn wounds, ventilator-associated pneumonia, urinary catheter-related infections, and cancers (1). Recognized as a notorious multidrug-resistant pathogen, *P. aeruginosa* poses a significant threat to global public health (2, 3). Expression of virulence and resistance genes is tightly regulated in *P. aeruginosa*. For instance, quorum-sensing (QS) system, a cell-to-cell communication system, is a critical system that regulates virulence gene expression in a cell density-dependent manner in *P. aeruginosa* (4).

*P. aeruginosa* has three well-characterized QS systems: two acyl-homoserine lactone (AHL)-based systems, Las and Rhl, and a quinolone-based system, Pqs (5). In the Las system, LasI synthesizes the autoinducer *N*-(3-oxododecanoyl)-L-homoserine lactone (3-oxo-C_12_-HSL), which is recognized by LasR. Similarly, in the Rhl system, RhlI produces *N*-butanoyl-L-homoserine lactone (C_4_-HSL), which is recognized by RhlR. The Pqs system utilizes 3,4-dihydroxy-2-heptylquinoline (PQS) and 4-hydroxy-2-heptylquinoline (HHQ) as autoinducers, which are synthesized by PqsABCD/PqsH and recognized by PqsR. These systems collectively regulate the expression of key virulence factors, such as protease, rhamnolipid, and pyocyanin (PYO), which are essential for the full virulence of *P. aeruginosa* (6–9). Among them, PYO, the redox-active phenazine molecule, is the most distinguishable one due to its blue-green color.

Three QS systems were thought to operate hierarchically, with the Las system at the top of the regulatory network (9). However, strains with loss-of-function mutations in *lasR* were frequently isolated, especially in strains isolated from chronic infections (10–15). Furthermore, the activity and hierarchy of QS systems are dynamic and influenced by various intracellular and extracellular factors. Increasing regulatory systems, including two-component systems and small RNAs, have been shown to modulate QS activity in response to diverse environmental cues (16–18). Despite these advances, molecular mechanisms underlying the regulation of QS activity and virulence gene expression remain poorly understood.

Nucleoid-associated proteins (NAPs) are small, abundant proteins in bacteria that play critical roles in genome architecture, metabolism, stress adaptation, and virulence (19). Among NAPs, the HU protein, typically composed of subunit α (HupA) and subunit β (HupB), modulates various physiological processes mainly with its non-specific DNA-binding activity (20). In this study, we isolated a PYO-hyperproducing *P. aeruginosa* isolate PA_HN008 and used this strain to explore novel genetic elements that modulate QS activity. Leveraging PYO production as a fast readout, we identified the α subunit of the HU protein, PA5348 (HupA), as a direct transcriptional activator of the Pqs system. Our findings provide new insights into the role of NAPs in activating the QS system and virulence factor production.

## RESULTS

### The *P. aeruginosa* isolate PA_HN008 displays hyperproduction of PYO

**Fig 1:**
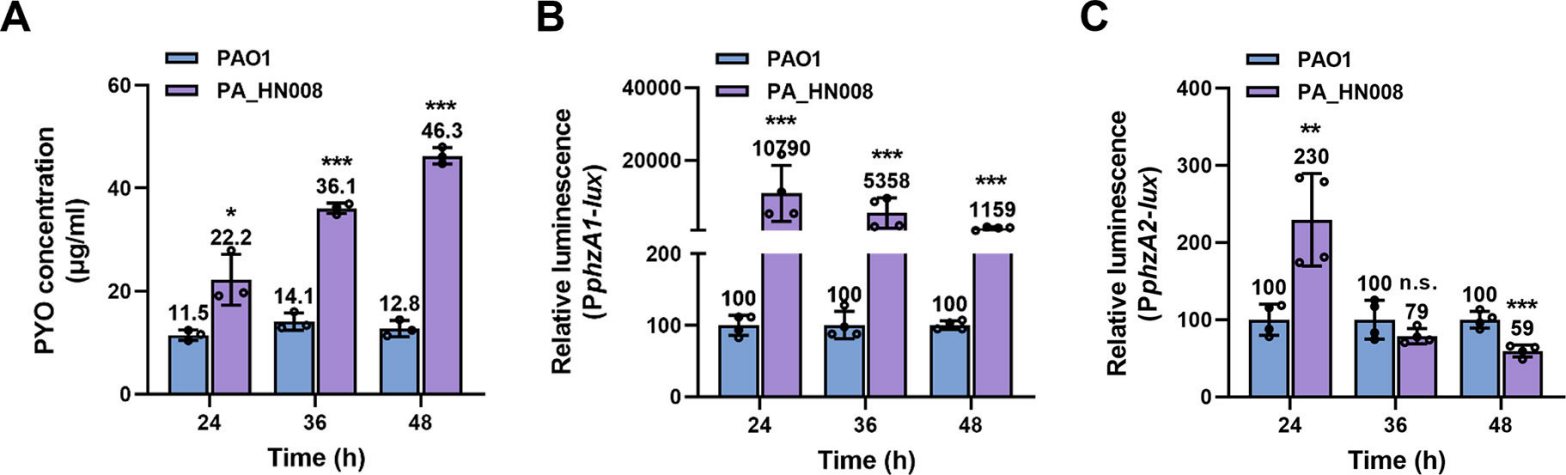
PA_HN008 displays hyperproduction of PYO. (**A**) PYO production was quantified in PAO1 and PA_HN008 after two strains were cultured statically at 37°C for 24, 36, and 48 h. (**B**) Relative activity of the *phz1* promoter in PA_HN008 compared to PAO1 after two strains were cultured for 24, 36, and 48 h. (**C**) Relative activity of the *phz2* promoter in PA_HN008 compared to PAO1 after two strains were cultured for 24, 36, and 48 h. Statistical significance was calculated based on Student’s *t*-test (n.s., not significant; *, *P* < 0.05; **, *P* < 0.01; and ***, *P* < 0.001).

We recently isolated the *P. aeruginosa* strain PA_HN008 from a urinary tract infection in Guangdong Women and Children Hospital. PA_HN008 produced substantially higher levels of PYO when it was statically cultured in liquid media after 24, 36, and 48 h compared to the laboratory reference strain PAO1 (Fig. 1A). PYO biosynthesis involves two nearly identical but differentially regulated *phz* operons, *phz1* (*phzA1B1C1D1E1F1G1*) and *phz2* (*phzA2B2C2D2E2F2G2*), as well as *phzM* and *phzS* genes (21, 22). Promoter activity of two operons was then measured, and the result showed that the activity of the P*phz1* promoter was 107.9-, 53.58-, and 11.59-fold higher in PA_HN008 than that in the PAO1 strain after two strains were statically cultured for 24, 36, and 48 h (Fig. 1B). In contrast, the activity of the P*phz2* promoter was only 2.3-fold higher in the PA_HN008 strain than that in the PAO1 strain when the two strains were statically cultured for 24 h, and the activity of P*phz2* in PA_HN008 became lower than that in PAO1 after the two strains were statically cultured for 36 and 48 h (Fig. 1C). These results implied that PYO hyperproduction in PA_HN008 was mainly ascribed to the hyperexpression of the *phz1* operon.

### PA_HN008 is a Las-null strain belonging to the clonal complex of MLST1239

To obtain the genetic information of the newly isolated PA_HN008 strain, we sequenced the genome of PA_HN008 using Illumina HiSeq and Nanopore sequencing platforms. As shown in Fig. S1A, the genome of PA_HN008 is 6,591,441 bp in length, which is larger than that of PAO1 (6,264,404 bp). The GC content of the PA_HN008 genome is 66.34%, and the genome is predicted to contain 6,050 protein-coding genes, 12 rRNAs, 63 tRNAs, 6 ncRNAs, 41 pseudogenes, 3 CRISPR arrays, and 7 prophages (Fig. S1B). PA_HN008 is assigned to the multiple locus sequence type (MLST) 1239, and phylogenetic analysis of PA_HN008 with 92 additional *P. aeruginosa* strains belonging to the same MLST1239 clonal complex, with their genomes publicly available in NCBI, showed that PA_HN008 is closely related to Pa608 and M18 (Fig. S1C). Interestingly, the geographic origins of PA_HN008, Pa608, and M18 are close, but Pa608 and M18 were isolated from environmental sources. Genome analysis revealed that the whole *lasR* gene and the neighboring *lasI* and *rsaL* genes are absent (Fig. S1D). We further searched for LasR and LasI mutations in strains belonging to the clonal complex of MLST1239 by aligning with the corresponding reference genes in PAO1 and found that 34.4% (32 out of 93) of strains carried mutations in LasR, a ratio slightly lower than that reported in different *P. aeruginosa* genome collections (12–14). In contrast, LasI is highly conserved because all LasI except that in PA_HN008 were found identical to that in PAO1 (Table S1).

### Hyperproduction of PYO in PA_HN008 relies on the activation of the RhlI-RhlR-PqsE pathway at the late growth stage

**Fig 2:**
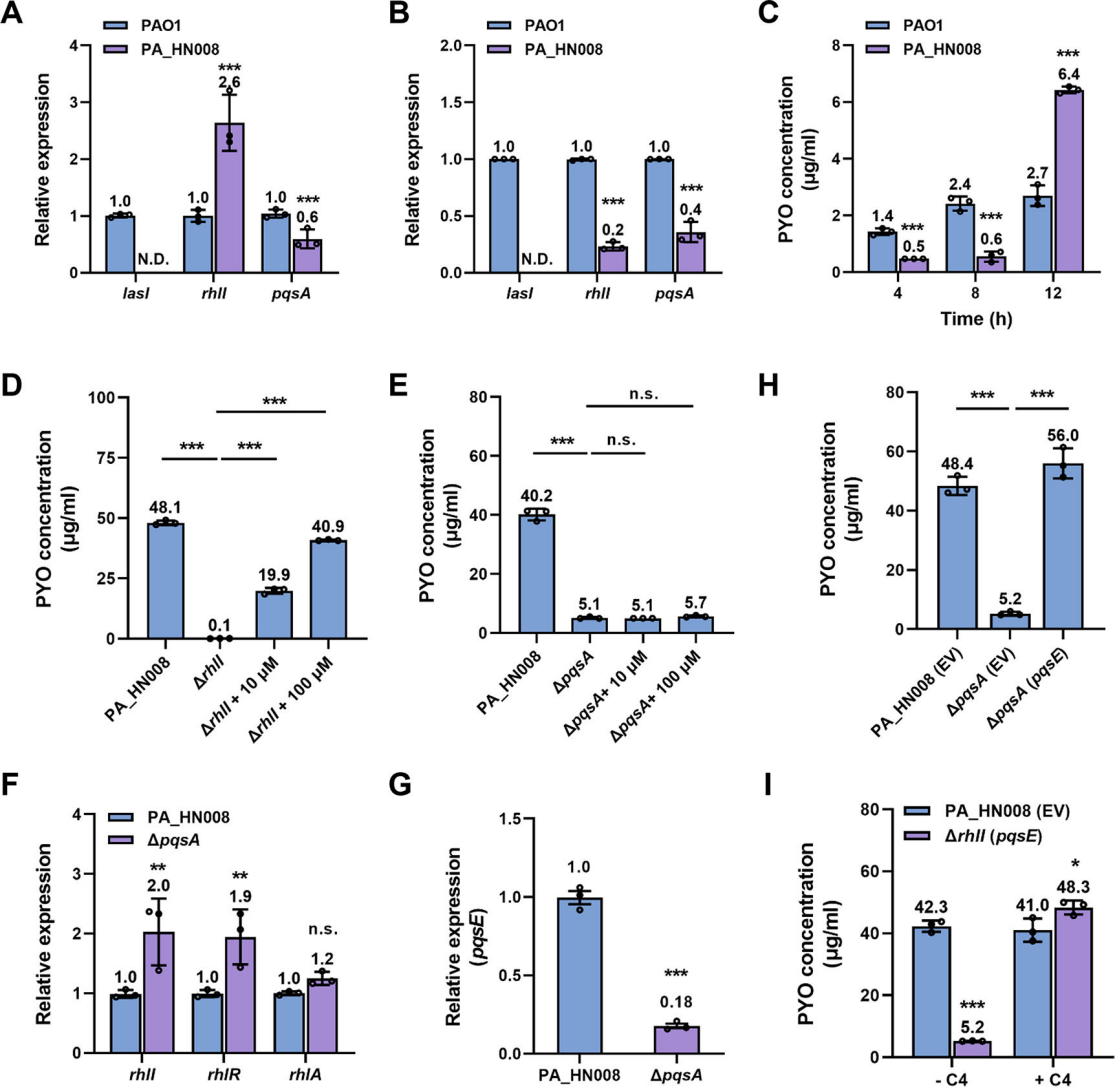
PYO biosynthesis in PA_HN008 is controlled by the RhlI-RhlR-PqsE pathway. (**A and B**) Relative expression of the *lasI*, *rhlI*, and *pqsA* genes in the PA_HN008 strain compared to the PAO1 strain when they were cultured for 24 h (A, late growth stage) or cultured till OD_600_ of 1.0 (B, the early exponential stage). N.D., not detected. (**C**) PYO production was quantified in PAO1 and PA_HN008 after two strains were cultured with agitation for 4, 8, and 12 h. (**D**) PYO production was quantified in the PA_HN008 Δ*rhlI* mutant when the mutant was cultured with or without the supplementation of different concentrations of C_4_-HSL in the growth medium. (**E**) PYO production was quantified in the PA_HN008 Δ*pqsA* mutant when the mutant was cultured with or without the supplementation of different concentrations of PQS in the growth medium. (**F**) Relative expression of the *rhlI*, *rhlR*, and *rhlA* genes in the Δ*pqsA* mutant compared to the PA_HN008 wild-type (WT) strain. (**G**) Relative expression of the *pqsE* gene in the Δ*pqsA* mutant compared to the PA_HN008 WT strain. (**H**) PYO production was quantified in the PA_HN008 Δ*pqsA* mutant when it overexpressed PqsE. (**I**) PYO production was quantified in the PA_HN008 Δ*rhlI* mutant when it overexpressed PqsE in the presence or absence of 100 μM C_4_-HSL (C4). Statistical significance was calculated based on one-way ANOVA or Student’s *t*-test (n.s., not significant; *, *P* < 0.05; **, *P* < 0.01; and ***, *P* < 0.001).

PYO is an important virulence factor under the control of QS systems in *P. aeruginosa* (23). We further compared the expression levels of *lasI*, *rhlI*, and *pqsA*—key genes for the biosynthesis of autoinducers in the Las, Rhl, and Pqs systems—between PA_HN008 and PAO1 to evaluate the activity of three QS systems. Unlike the undetectable expression of *lasI* and a slightly lower expression level of *pqsA*, the reverse transcription-quantitative PCR (RT-qPCR) assay showed a higher expression level of *rhlI* in PA_HN008 than that in PAO1 after the two strains were cultured for 24 h with agitation (Fig. 2A). This result suggested that, although the Las system is absent, the highly active Rhl system in PA_HN008 potentially explains its hyperproduction of PYO. Given that LasR mutants are known to accumulate high levels of PYO during the late stationary phase (13), we next compared the expression of *rhlI* and *pqsA* genes in PA_HN008 and PAO1 strains at the exponential phase (OD_600_ = 1.0) and PYO production at the early growth stages when the two strains were cultured for 4 and 8 h with agitation. It was shown that the expression of *rhlI* and the production of PYO were lower in PA_HN008 than PAO1 at the early growth stage (Fig. 2B and C), indicating that the activation of the Rhl system contributes to the high level of PYO production at the late growth stage. To confirm if the Rhl system controls PYO production in PA_HN008, we deleted *rhlI* to disrupt the system. The result showed that deletion of *rhlI* substantially reduced PYO production, while exogenous supplementation of the autoinducer C_4_-HSL restored PYO production in the Δ*rhlI* mutant (Fig. 2D). These results demonstrated that PA_HN008 is a Las-null isolate with Rhl-dependent hyperproduction of PYO at the late growth stage, which showed a similar regulatory pattern of PYO production as common LasR mutants.

We then evaluated the involvement of the Pqs system, especially PqsE, in the PYO production and its connection with the Rhl system (24, 25). As expected, deletion of *pqsA* led to a dramatic decrease in PYO production in PA_HN008 (Fig. 2E), suggesting that the Pqs system is essential for PYO biosynthesis as well. However, possibly due to the full abolishment of the Pqs system after *pqsA* deletion, exogenous supplementation of the autoinducer PQS did not restore PYO production in the Δ*pqsA* mutant (Fig. 2E). We also found that deletion of the *pqsA* gene did not reduce *rhl* gene expression (Fig. 2F). Unlike inducing the Rhl system by the Pqs system in other *P. aeruginosa* strains (26), expression of *rhlI* and *rhlR* was slightly upregulated in the Δ*pqsA* mutant, suggesting that reduced PYO production in the Δ*pqsA* mutant was not due to the repression of the Rhl system. Increasing studies have reported that PYO production requires PqsE, which forms a regulatory complex with RhlR and its autoinducer C_4_-HSL (27–30). Since the deletion of *pqsA* led to a dramatic downregulation of *pqsE* (Fig. 2G), we next investigated whether PqsE participates in PYO production. Upon overexpressing PqsE in the Δ*pqsA* mutant, we found that PYO production was successfully restored (Fig. 2H). To further determine if PqsE mediates Rhl-dependent PYO production, we overexpressed PqsE in the Δ*rhlI* mutant but did not observe elevated PYO production in this strain (Fig. 2I). Interestingly, exogenous supplementation of C_4_-HSL during the growth of the Δ*rhlI* mutant with PqsE overexpression restored PYO production and even resulted in a level exceeding that of the wild-type (WT) strain (Fig. 2I), demonstrating that PYO production in PA_HN008 is controlled by the RhlI-RhlR-PqsE pathway.

### A genome-wide screening identifies a novel regulator, PA5348, of PYO production

**Fig 3:**
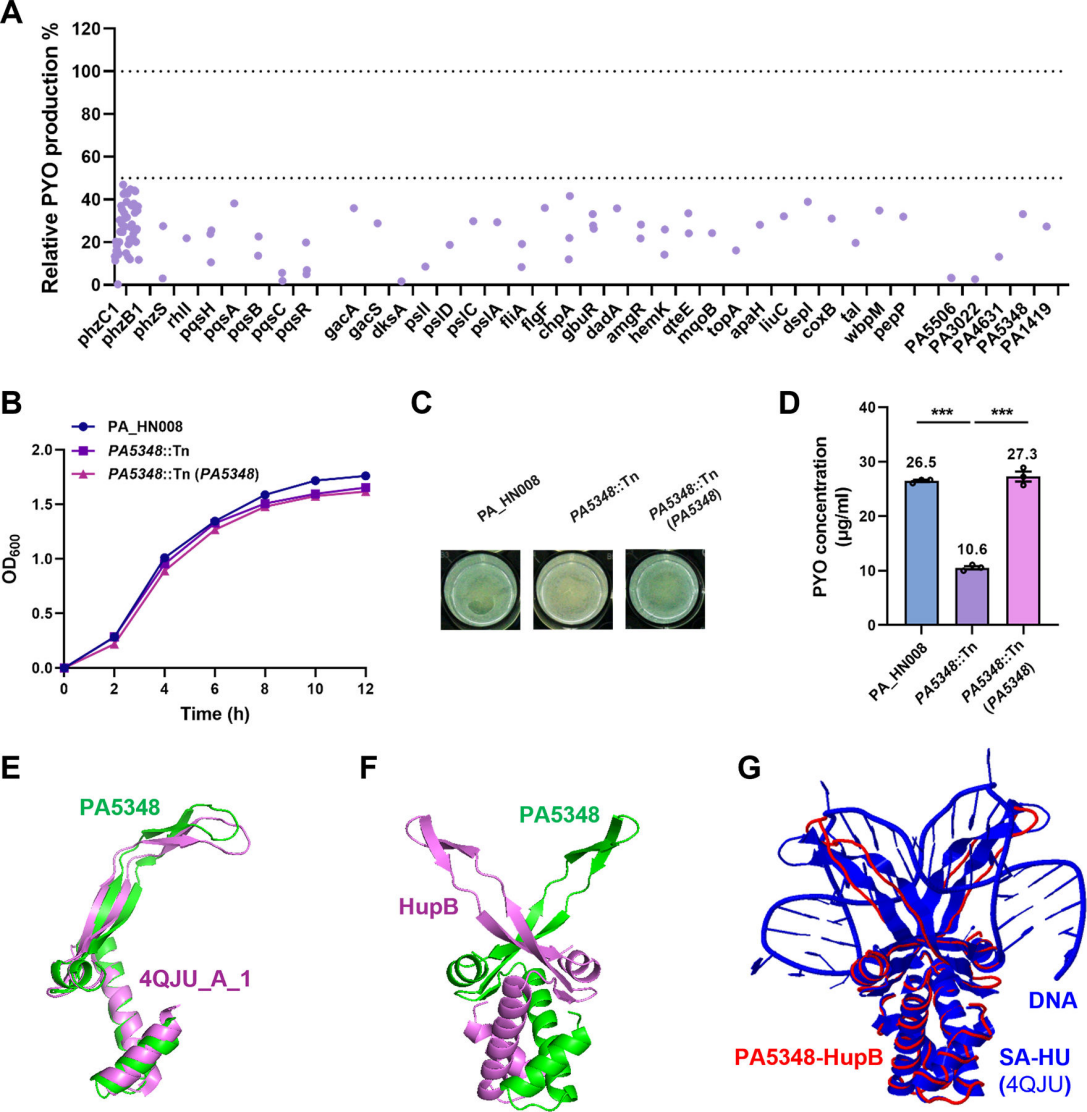
Identification of a novel regulator PA5348 (HupA) for PYO production. (**A**) One hundred one transposon-inserted mutants with more than 50% reduced production of PYO compared to the PA_HN008 WT strain. (**B**) Growth curves of the PA_HN008 WT, *PA5348*::Tn, and *PA5348*::Tn (*PA5348*) strains. (**C**) Color of the bacterial culture of PA_HN008 WT, *PA5348*::Tn, and *PA5348*::Tn (*PA5348*) strains. (**D**) PYO production was quantified in PA_HN008 WT, *PA5348*::Tn, and *PA5348*::Tn (*PA5348*) strains. (**E**) Structure alignment between PA5348 and the α subunit of the nucleoid-associated protein HU (4QJU_A_1) in *Staphylococcus aureus*. (**F**) Predicted interaction between PA5348 and HupB in *P. aeruginosa*. (**G**) Structure alignment between PA5348-HupB complex and HupA-HupB complex (HU protein, 4QJU) in *S. aureus*. Statistical significance was calculated based on one-way ANOVA (***, *P* < 0.001).

Due to the hyperproduction of the QS-controlled virulence factor PYO in PA_HN008, we next moved to screen genetic requirements controlling PYO production in this strain to identify novel modulators of QS in *P. aeruginosa*. Transposon mutagenesis was performed, and mutants that displayed reduced or abolished PYO production were selected. We generated 26,217 mutants in total, with 529 of them showing reduced PYO production as evaluated by the color of cell cultures during the primary screening. Next, 529 PA_HN008 mutants were cultured for PYO quantification, and finally, transposon insertion sites of 101 mutants, which displayed more than 50% reduced production of PYO compared to the PA_HN008 WT strain, were determined by arbitrary PCR and Sanger sequencing. As a result, a large proportion of mutants (63/101) showed the disruption of PYO biosynthetic genes (51 insertions in *phz* genes) and the Rhl and Pqs systems (1 insertion in *rhlI* and 11 insertions in *pqs* genes) (Fig. 3A). This result further confirmed that both Rhl and Pqs systems are essential for PYO production in PA_HN008.

In addition to PYO biosynthetic genes and QS genes, we were particularly interested in the transposon insertions located in uncharacterized genes. Five genes encoding PA5506 (HTH rpiR-type domain-containing protein), PA3022 (MOSC domain-containing protein), PA4631 (NAD-dependent epimerase/dehydratase domain-containing protein), PA5348 (DNA-binding protein HU-alpha), and PA1419 (probable transporter) were screened out with potential roles in regulating PYO production. In this study, we focused on one of the five uncharacterized genes, the DNA-binding protein PA5348, to explore how it controls PYO production. The growth of *P. aeruginosa* was not influenced by the mutation of PA5348 (Fig. 3B), and the function of PA5348 in regulating PYO production was further verified by the complementation of PA5348 in the mutant (Fig. 3C and D). Protein structure alignment using AlphaFold (31) showed that PA5348 shares high similarity with the α subunit of the nucleoid-associated protein HU (4QJU_A_1) in *Staphylococcus aureus* (Fig. 3E). Since HU protein is generally a heterocomplex, which is composed of α and β subunits, and PA1804 (HupB) has been identified as the β subunit in *P. aeruginosa* (32), we next employed DMFold to evaluate the interaction between PA5348 and HupB proteins (33). Interaction between PA5348 and HupB was predicted, and the PA5348-HupB complex showed high similarity with the HU (HupA-HupB) complex (4QJU) in *S. aureus* (Fig. 3F and G). Owing to the high similarity between PA5348 and HupA in *S. aureus,* as well as the potential formation of the PA5348-HupB complex in *P. aeruginosa*, we hereafter refer to PA5348 as HupA.

### HupA mainly regulates the production of PYO, but not other important virulence factors

**Fig 4:**
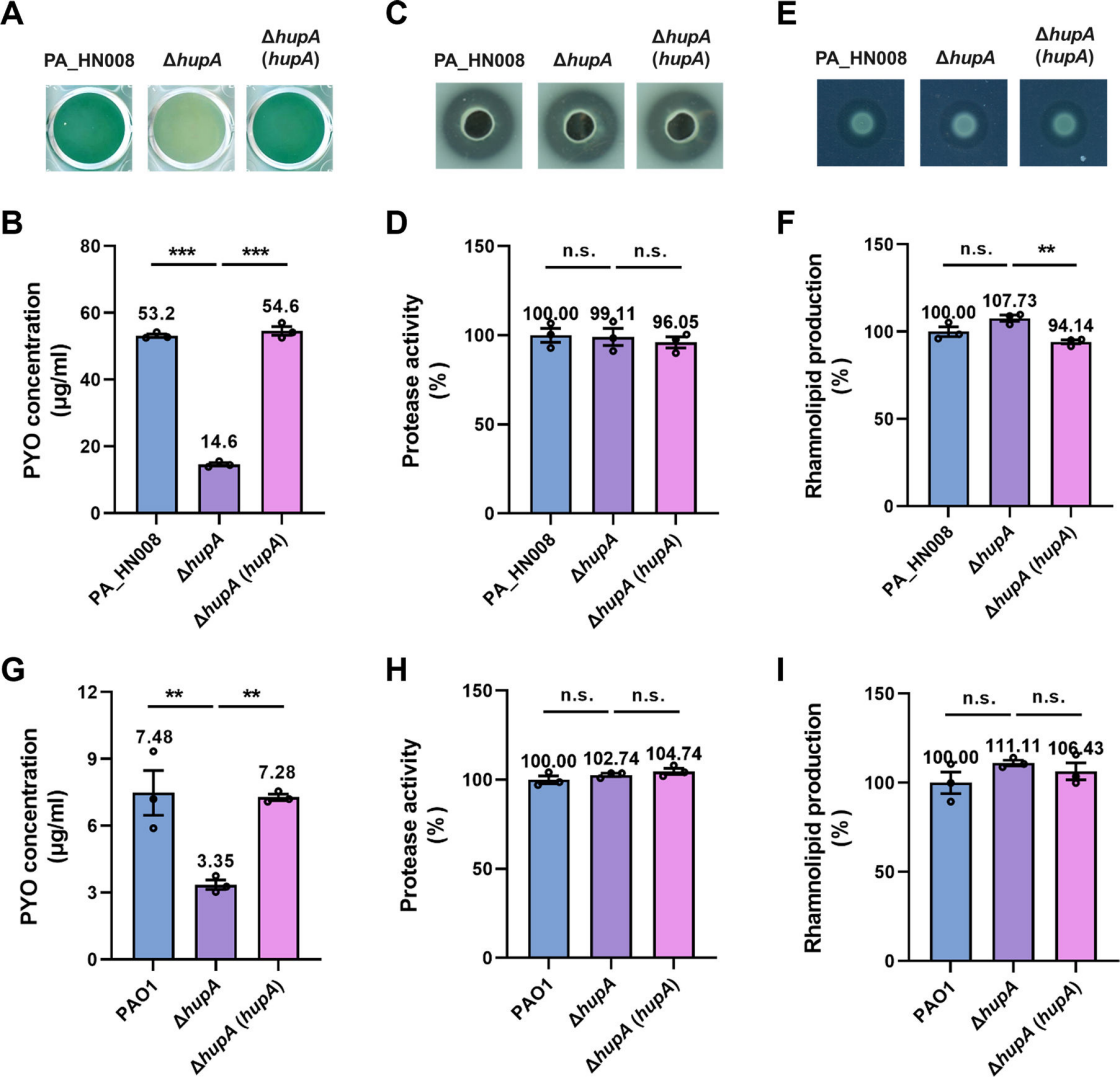
HupA regulates the production of PYO but not protease and rhamnolipid in *P. aeruginosa*. (**A**) Color of the bacterial culture of PA_HN008 WT, Δ*hupA*, and Δ*hupA* (*hupA*) strains. (**B**) PYO production was quantified in PA_HN008 WT, Δ*hupA*, and Δ*hupA* (*hupA*) strains. (**C and D**) Protease activity was measured in PA_HN008 WT, Δ*hupA*, and Δ*hupA* (*hupA*) strains. (**E and F**) Rhamnolipid production was measured in PA_HN008 WT, Δ*hupA*, and Δ*hupA* (*hupA*) strains. (**G–I**) PYO (**G**), protease (**H**), and rhamnolipid (**I**) were measured in PAO1 WT, Δ*hupA*, and Δ*hupA* (*hupA*) strains. Statistical significance was calculated based on one-way ANOVA (n.s., not significant; **, *P* < 0.01; ***, *P* < 0.001).

We next generated the mutant Δ*hupA* and the gene-complemented strain Δ*hupA*(*hupA*) to further understand the role of HupA in regulating the production of virulence factors. First, consistent with the transposon mutant *PA5348*::Tn, the gene deletion mutant Δ*hupA* showed a substantial decrease in PYO production compared to the WT and Δ*hupA*(*hupA*) strains (Fig. 4A and B). In addition to PYO, we measured the production of two other important QS-controlled virulence factors, protease and rhamnolipid (9). Unlike PYO, the production of protease and rhamnolipid was not influenced by the deletion of *hupA* (Fig. 4C through F). Since HupA was predicted to interact with the HupB subunit to form the HU complex, we further constructed the double-deletion mutant Δ*hupA*Δ*hupB* and found that complementation of *hupA* in the double-deletion mutant was unable to restore PYO production (Fig. S2), suggesting that HupA regulates PYO production in the HupB-dependent manner. Given that the contribution of HupA to regulate PYO production was found in the newly isolated strain PA_HN008, we further examined the function of HupA in the PAO1 strain. Similar to the PA_HN008 strain, deletion of *hupA* in PAO1 only reduced the production of PYO, while it did not impact the production of protease and rhamnolipid (Fig. 4G through I). In addition, we analyzed the presence and similarity of HupA in 608 *P. aeruginosa* genomes collected from the Pseudomonas Genome Database (https://www.pseudomonas.com/) and found that HupA is highly conserved, with a minimal similarity of 97.8%. Altogether, HupA is potentially a conserved positive regulator of PYO production in *P. aeruginosa*.

### HupA induces PYO production partially through the activation of the Pqs system

**Fig 5:**
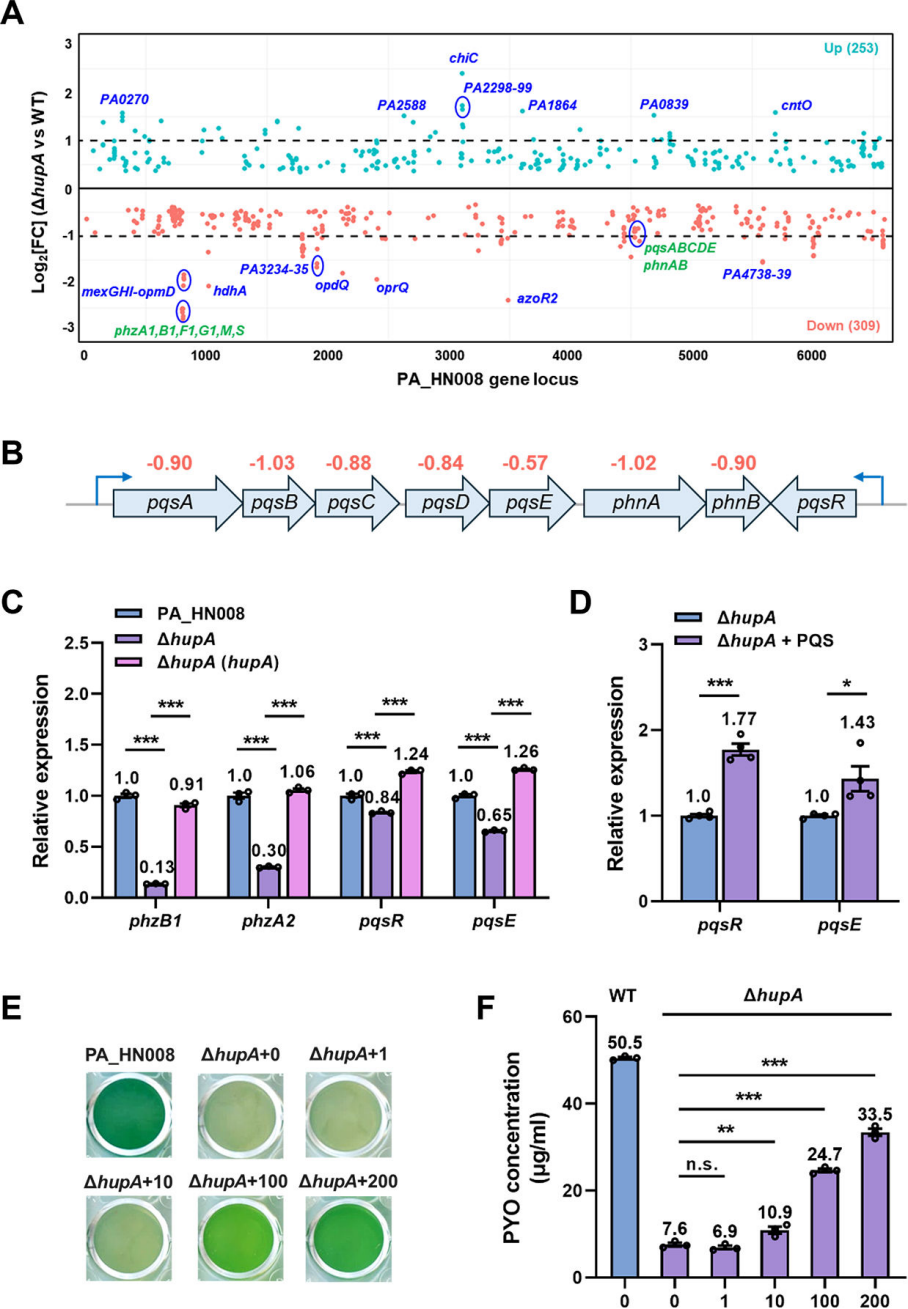
HupA regulates PYO production partially through the Pqs system. (**A**) Genome-wide transcriptomic profiles of the PA_HN008 WT and Δ*hupA* strains. Green dots were the genes upregulated in the Δ*hupA* mutant, while orange dots were the genes downregulated in the Δ*hupA* mutant. Genes with dramatic differences in expression were selectively labeled. (**B**) The gene cluster involved in PQS biosynthesis is shown. Values above gene names indicate the log2 fold changes of gene expression in the Δ*hupA* mutant relative to that in the WT strain. (**C**) Relative expression of the *phzB1*, *phzA2*, *pqsR*, and *pqsE* genes in the PA_HN008 Δ*hupA* mutant compared to the WT strain. (**D**) Relative expression of the *pqsR* and *pqsE* genes in the PA_HN008 Δ*hupA* mutant with or without the supplementation of 100 μM PQS during growth. (**E**) Color of the bacterial culture of PA_HN008 Δ*hupA* mutant when grown with the supplementation of different concentrations of PQS (μM) in the medium. (**F**) PYO production was quantified in the PA_HN008 Δ*hupA* mutant when grown with the supplementation of PQS (μM) in the medium. Statistical significance was calculated based on one-way ANOVA or Student’s *t*-test (n.s., not significant; *, *P* < 0.05; **, *P* < 0.01; and ***, *P* < 0.001).

To further understand the regulatory mechanisms of PYO production by the HupA protein, we conducted RNA sequencing (RNA-seq) and compared the transcriptomic profiles between the WT and Δ*hupA* strains. A total of 253 genes were upregulated, and 309 genes were downregulated with the adjusted *P* value <0.05 in the mutant Δ*hupA* compared to the WT strain (Fig. 5A; Table S2). Among them, only 39 genes and 63 genes were upregulated and downregulated with the log2 fold change above 1.0, respectively (Table S2). Consistent with the reduction of PYO production in Δ*hupA*, the RNA-seq result showed that *phz1*, *phzM*, and *phzS* genes were downregulated in the mutant Δ*hupA*, while genes involved in producing other virulence factors, such as protease and rhamnolipid, were not found (Fig. 5A).

Consistent with the pattern that PYO production in PA_HN008 depends on the RhlI-RhlR-PqsE pathway, genes in the *pqs* operon were significantly downregulated in the mutant Δ*hupA* compared to the WT strain. In detail, *pqsA*, *pqsB*, *pqsC*, *pqsD*, and *pqsE* were downregulated with log2 fold changes of 0.90, 1.03, 0.88, 0.84, and 0.57, respectively (Fig. 5B), suggesting that the reduced PYO production in the mutant Δ*hupA* was possibly ascribed to the decreased activity of the Pqs system and, more specifically, the reduced expression of PqsE. RT-qPCR verified the downregulation of *phz* genes as well as the *pqsR* and *pqsE* genes (Fig. 5C). Since PQS is the product of the *pqs* operon and can also positively regulate the expression of the *pqs* operon (34), we then exogenously supplemented PQS during the growth of the mutant Δ*hupA*. The expression of *pqsE* and PYO production of the mutant were elevated as expected (Fig. 5D through F), indicating that the Pqs system remains functional in the Δ*hupA* mutant, and HupA positively regulates PYO production by activating the Pqs system, specifically through the PqsE protein, as it plays the key role in controlling PYO production. Notably, deletion of *hupA* slightly reduced the expression of *pqs* genes, and supplementation of PQS partially restored PYO production even if the concentration of PQS was quite high, suggesting the presence of additional pathways that mediate HupA’s regulation of PYO production.

### HupA directly controls the expression of both *pqs* genes and PYO biosynthetic genes

**Fig 6:**
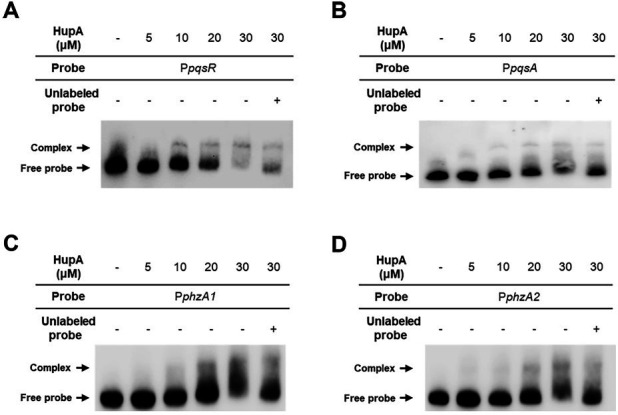
HupA binds to the promoters of *pqs* and *phz* genes. EMSAs showed a clear interaction between HupA with the promoter of *pqsR* (**A**) and a weak interaction between HupA with promoters of *pqsA* (**B**), *phzA1* (**C**), and *phzA2* (**D**).

Considering that HupA is a DNA-binding protein, we tried to examine whether HupA regulates *pqs* gene expression by directly interacting with its promoters. We purified the HupA protein and performed electrophoretic mobility shift assays (EMSAs). It was shown that the presence of HupA led to a clear shift of the *pqsR* promoter and a slight shift of the *pqsA* promoter (Fig. 6A and B), meaning that HupA can form complexes with *pqsA* and *pqsR* promoters. As a control, HupA did not form a complex with the *recA* promoter (Fig. S3). These results indicated that HupA specifically recognizes and binds to *pqsA* and *pqsR* promoters. Since HupA regulates PYO production partially through the Pqs system, we further examined whether HupA binds to the promoters of PYO biosynthetic genes. Interestingly, EMSAs showed weak interactions between HupA and promoters of the *phzA1* and *phzA2* genes (Fig. 6C and D). Altogether, these results indicate that HupA positively regulates PYO production through activating the transcription of both *pqs* genes and PYO biosynthetic genes.

## DISCUSSION

QS is a mechanism of bacterial cell-to-cell communication, which controls the expression of genes encoding virulence factors in a cell density-dependent manner. *P. aeruginosa* has three hierarchically organized QS systems, Las, Rhl, and Pqs. However, *P. aeruginosa* strains carrying LasR mutations are frequently isolated from diverse environments (13). In this study, we isolated a Las-null but Rhl- and Pqs-active clinical strain, PA_HN008, and demonstrated that hyperproduction of PYO in PA_HN008 was due to the active Rhl and Pqs systems at the late growth stage. QS activity is fine-tuned by diverse endogenous and exogenous factors, and the regulation is extremely complicated and remains largely unexplored. Using the PYO-hyperproducing strain PA_HN008, we constructed a mutant library to comprehensively screen genetic elements that regulate QS activity using the green-blue virulence factor PYO as a fast readout. As a result, we identified a DNA-binding protein HupA, encoding the α subunit of the HU protein, which positively regulates PYO production by specifically and directly activating the transcription of *pqs* genes and PYO biosynthetic genes. Although the contribution of HupA to PYO production was identified using the clinical isolate PA_HN008, HupA was found to be highly conserved in *P. aeruginosa*, which suggests that HupA could be a universal modulator of Pqs activity and PYO production in *P. aeruginosa*.

Unlike other NAPs such as Fis or H-NS, which often exert broad regulatory effects on global gene expression in diverse bacterial species (35, 36), we show that the gene regulatory spectrum of HupA is relatively narrow. Loss of HupA only led to 39 upregulated genes and 63 downregulated genes with log2 fold change above 1.0. Particularly, we found that HupA modulates the transcription of *pqs* and *phz* genes by specifically binding to their promoters, although the binding details have not been determined. The interactions between HupA and the promoters, especially the *pqsA* and *phz* promoters, were not very strong. These results not only broaden our knowledge about the regulatory specificity of NAPs, which typically regulate various virulence-related pathways, but also answer why deletion of *hupA* did not influence the production of other QS-controlled virulence factors such as protease and rhamnolipid. PYO production is mainly regulated by the Rhl and Pqs systems, while the production of proteases and rhamnolipids is primarily regulated by the Las and Rhl systems, respectively, instead of the Pqs system (37).

HU is one of the most abundant and conserved NAPs in bacteria (19). Although it is an important protein essential for various physiological processes, such as DNA compaction, replication, transcription, recombination, and shape modulation, it binds DNA generally in a sequence-nonspecific manner with high affinity to abnormal DNA structures during DNA damage (38). Regulation of bacterial virulence by HU proteins was reported in several bacterial species (39–41), but the detailed regulatory mechanisms remain elusive. Owing to its DNA-binding ability, it was found that HU protein can bind to the double-stranded DNA to protect them from oxidative stress and thus elevate the virulence of *Francisella tularensis* (42). Interestingly, HU protein is also reported to specifically bind to target gene promoters. For example, direct binding of HU proteins to the promoters of type VI secretion system genes was reported in *Enterobacter cloacae* (43). In this study, the direct binding of HupA to the *pqs* and *phz* promoters, as shown by EMSA, further underscores the role of the HU subunit as a sequence-specific transcriptional activator, a function distinct from the canonical non-specific DNA-binding activity of HU proteins. This dual functionality positions HupA as a unique player in bridging genome architecture and precise gene regulation. However, the mechanism by which HupA interacts with HupB to modulate PYO production remains unclear. The physiological functions of HupB and the HU complex in *P. aeruginosa* also deserve further investigation.

In summary, this study uncovers PA5348 (HupA) as a novel regulator controlling QS activity and virulence gene expression in *P. aeruginosa*. By elucidating its positive regulation on PYO production through specific activation of the *pqs* and *phz* genes, we provide new insights into the function of the α subunit of the HU protein in directly modulating target gene transcription.

## MATERIALS AND METHODS

### Bacterial strains, plasmids, primers, and growth conditions

Bacterial strains, plasmids, and primers are listed in Table S3. *P. aeruginosa* PA_HN008 was isolated from a mid-stream urine sample of a 3-year-old pediatric patient in Guangdong Women and Children Hospital (Guangzhou, China). Bacterial strains were routinely cultured in LB medium (10 g/L tryptone, 5 g/L yeast extract, and 5 g/L NaCl) at 37°C with 220 rpm agitation or in static. Antibiotics were added when required: gentamicin, 50 μg/mL; tetracycline, 50 μg/mL for *P. aeruginosa*.

### Gene deletion and gene complementation

Gene deletion in PA_HN008 was conducted according to the previously reported method (44). Briefly, upstream and downstream sequences of the target gene were amplified by PCR using the genomic DNA of PA_HN008 as the template and then cloned into the suicide vector pK18mobsacB between the *Bam*HI and *Hin*dIII sites. The constructed vector was introduced into PA_HN008 by tri-parental mating with the helper plasmid pRK2013. Desired gene deletion mutants were selected using LB agar plates containing 10% sucrose and verified by PCR and Sanger sequencing. For gene complementation in the mutant, the DNA fragment containing the coding region of the target gene and its native promoter was amplified by PCR using the genomic DNA of PA_HN008 as the template and then cloned into the plasmid of mini-CTX-*lacZ*. After conjugation with the *E. coli* SM10 strain, the DNA fragment containing the target gene was integrated into the genome of the gene deletion mutant. Chromosomal integration of the target gene was examined by PCR and Sanger sequencing.

### Construction of promoter-*lux* reporter and promoter activity assay

Promoter sequences were amplified using the genomic DNA of PA_HN008 or PAO1 as the template and then cloned into the promoter-less vector mini-CTX-*lux*. The CTX-promoter-*lux* fusions were integrated into the genomes of *P. aeruginosa* strains by conjugation. Overnight cultures of *P. aeruginosa* strains containing promoter-*lux* fusions were adjusted to an OD_600_ of 1.0 and then diluted 1:50 into fresh LB medium. Luminescence and OD_600_ were monitored using a microplate reader (BioTek, USA) after the strains were cultured for 24, 36, or 48 h.

### Genome sequencing, assembly, and bioinformatic analyses

The genomic DNA of PA_HN008 was extracted using the EasyPure Genomic DNA Kit (TransGen, China). Short reads and long reads were sequenced with an Illumina HiSeq platform (Sangon, China) and a Nanopore MinION sequencer (Oxford Nanopore Technologies, UK), respectively. Quality-based filtering and adapter trimming were performed using Trimmomatic version 0.39 (45) and NanoFilt version 2.8.0 (46). The genome was hybrid assembled using Unicycler version 0.50 (47). The quality of the assembled genome was evaluated using QUAST version 5.0.2 (48). MLST of the genome of PA_HN0008 was determined using PubMLST (49). To achieve the phylogenetic position of PA_HN0008 within the group of MLST1239 genomes, all genomes affiliated with MLST1239 and their BioSample information were retrieved from NCBI (Table S1). In total, a data set of 93 genomes was curated and qualified using CheckM version 1.2.2 (50). All genomes were annotated using Prokka version 1.14.5 with a database curated for the genus *Pseudomonas* from all annotated genomes deposited at GenBank (51). All these 93 genomes passed the criteria of high-quality genomes (completeness ≥ 95% and contamination ≤ 5%). A core genome of these 93 genomes was called, and SNPs were harvested from the alignment using ParSNP version 1.1.2 (52). The phylogenetic tree was built using IQ-TREE version 2.2.0 with the best model proposed by ModelFinder and visualized using iTOL (53).

### RNA-seq

Overnight cultures of *P. aeruginosa* strains were adjusted to an OD_600_ of 1.0 and then diluted 1:50 into fresh LB medium. After 24-h growth with agitation at 220 rpm, 0.5 mL cell culture was then collected, and total RNA was extracted using the Eastep Super Total RNA Extraction Kit (Promega, USA). Library construction and high-throughput sequencing were conducted using the Illumina platform at Frasergen (Wuhan, China). Quality control of raw reads was conducted using Trimmomatic version 0.32 (45). Mapping of qualified reads was performed against the genome of PA_HN008 using BWA version 0.7.17-r1188 (54), SAMtools version 1.15 (55), and bamkeepgoodreads of Stampy version 1.0.32 (56). featureCounts of Subread version 2.0.3 (57) was used to generate a count matrix, which was submitted to DESeq2 (58) for identifying DEGs.

### Reverse transcription-quantitative PCR

Except that cells were collected at the exponential phase (OD_600_ = 1.0) for gene expression analysis in Fig. 2B, bacterial culture conditions were the same as those described in the RNA-seq experiment. One milliliter of bacterial culture was collected, and total RNA was extracted using the Eastep Super Total RNA Extraction Kit (Promega, USA) following the manufacturer’s instructions. cDNA was reverse transcribed using the FastKing gDNA Dispelling RT SuperMix (TIANGEN, China). qPCR was performed using the ChamQ Universal SYBR qPCR Master Mix (Vazyme, China) in an ABI QuantStudioTM6 Flex system. The *recA* gene was used as the internal reference gene, and the 2^-ΔΔCt^ method was used to calculate the relative expression of the target genes (59).

### PYO quantification

PYO production was quantified based on the protocol described previously with slight modifications (60). Overnight cultures of *P. aeruginosa* strains were adjusted to an OD_600_ of 1.0 and then diluted 1:50 into fresh LB medium. Except for the bacterial culture conditions indicated in the legends of Fig. 1A and 2C, bacteria were generally cultured for 36 h in static. One milliliter of liquid *P. aeruginosa* culture was centrifuged at 13,000 rpm for 5 min. A volume of 750 μL of supernatant was then collected and mixed with 450 μL of chloroform by vortexing. After centrifugation at 13,000 rpm for 5 min, 400 μL liquid of the chloroform phase was collected and subsequently mixed with 200 μL of 0.2 M HCl. A volume of 100 μL of liquid from the aqueous phase was collected, and its absorbance at 510 nm was measured. The concentration of PYO was determined according to the standard curve.

### Rhamnolipid measurement

Rhamnolipid was semi-quantitatively measured according to the previously described protocol with slight modification (61). The overnight culture of *P. aeruginosa* was adjusted to OD_600_, and 2 μL bacterial culture was collected and spotted on the CTAB-methylene blue agar plates. After the plates were incubated for 48 h at 37°C, the diameters of halos were measured. The relative diameters were used to indicate the relative production of rhamnolipid.

### Protease activity assay

A plate assay was used to measure the protease activity of *P. aeruginosa*. Overnight culture of *P. aeruginosa* was adjusted to an OD_600_ of 1.0 and then diluted 1:100 into 5 mL of fresh LB medium. After incubation at 37°C for 24 h, culture supernatant was collected by centrifugation. Two microliters of culture supernatant was spotted on nonfat-dried milk plates. The plates were incubated at 37°C for 12 h, and the area of each proteolytic halo was measured to evaluate the relative proteolytic activity.

### Purification of HupA

The coding region of HupA was amplified from the PA_HN008 genome and cloned into the pGEX plasmid, generating the new plasmid pGEX-*hupA*. pGEX-*hupA* was transformed into *E. coli* BL21(DE3), and the strain was incubated for 16 h. The overnight culture was diluted 1:100 into 1 L of fresh LB medium. Expression of the HupA protein was induced with 0.5 mM isopropyl β-D-1-thiogalactopyranoside at 16°C for 16 h. Protein purification was conducted using Glutathione Beads (Smart-Lifescience, China), and purified proteins were verified by sodium dodecyl-sulfate-polyacrylamide gel electrophoresis.

### EMSA

Promoter regions were amplified using primers listed in Table S3 and labeled with biotin utilizing the Biotin 3′ End DNA Labeling Kit (Thermo Fisher Scientific, USA). Protein-DNA interactions were analyzed with the LightShift Chemiluminescent EMSA Kit (Thermo Fisher Scientific, USA). Briefly, the biotin-labeled probes were incubated in binding buffer with varying concentrations of HupA at 25°C for 30 min. The reaction mixtures were combined with 5× Loading Buffer and then separated on a native polyacrylamide gel via electrophoresis. The DNA probes were subsequently transferred to a nylon membrane and crosslinked under UV irradiation. The membranes were sequentially washed with Blocking Buffer, Wash Buffer, and Substrate Equilibration Buffer. Finally, DNA and protein-DNA complexes were visualized by chemiluminescence using the Working Substrate Solution and captured with a cooled CCD camera (Tanon, China).

### Transposon mutagenesis

The *E. coli* SM10 strain containing the pBT20 plasmid was cultured overnight in LB medium at 37°C with 220 rpm agitation, while PA_HN008 was cultured overnight in LB medium at 42°C with 220 rpm agitation. Cell densities of the overnight cultured *E. coli* SM10 and PA_HN008 were determined by measuring OD_600_, and then *E. coli* SM10 and PA_HN008 were mixed at a cell ratio of 1.5 × 10^9^ and 0.5 × 10^9^, respectively. The mixture was pelleted and resuspended in 50 μL of LB medium, followed by spotting on the surface of an LB agar plate. Mating (pBT20 delivery from SM10 to PA_HN008) occurred during the incubation of the mixture at 37°C for 6–8 h. The mixture was scraped from the agar plate and resuspended in 300 μL of PBS buffer. One hundred microliters of cell suspension was serially diluted and spread on the VBMM plates with supplementation of 50 μg/mL gentamicin, and plates were incubated at 37°C for 16 h. Single colonies carrying various transposon insertions in the genome were cultured in static conditions in 100 μL of LB medium in 96-well plates for 36 h. Mutants with decreased PYO concentration were collected, and their identities were determined by arbitrary PCR and Sanger sequencing (62).

## Data Availability

The genomic data of PA_HN008 were deposited in the NCBI under the project accession number PRJNA899139. The transcriptomic data were deposited in the NCBI under the project accession number PRJNA1233608.

## SUPPLEMENTAL MATERIAL

Supplemental figures aem.02425-25-s0001.pdf Fig. S1 to S3.

Table S1 aem.02425-25-s0002.xlsx Genomes of 93 *P. aeruginosa* strains belonging to the clonal complex of MLST1239.

Table S2 aem.02425-25-s0003.xlsx Differentially expressed genes in the Δ*hupA* mutant compared to the PA_HN008 WT strain.

Table S3 aem.02425-25-s0004.xlsx Bacterial strains, plasmids, and primers used in this study.

ASM does not own the copyrights to Supplemental Material that may be linked to, or accessed through, an article. The authors have granted ASM a non-exclusive, world-wide license to publish the Supplemental Material files. Please contact the corresponding author directly for reuse.
