# Supplementary material for: The nucleoid-associated protein subunit HupA positively regulates the Pqs system and pyocyanin production in Pseudomonas aeruginosa
Source: Appl Environ Microbiol. 2026 Mar 9;92(4):e02425-25. doi: 10.1128/aem.02425-25 (PMC13101494; doi:10.1128/aem.02425-25)
Supplement: Supplemental figures — Fig. S1 to S3. [file aem.02425-25-s0001.pdf]

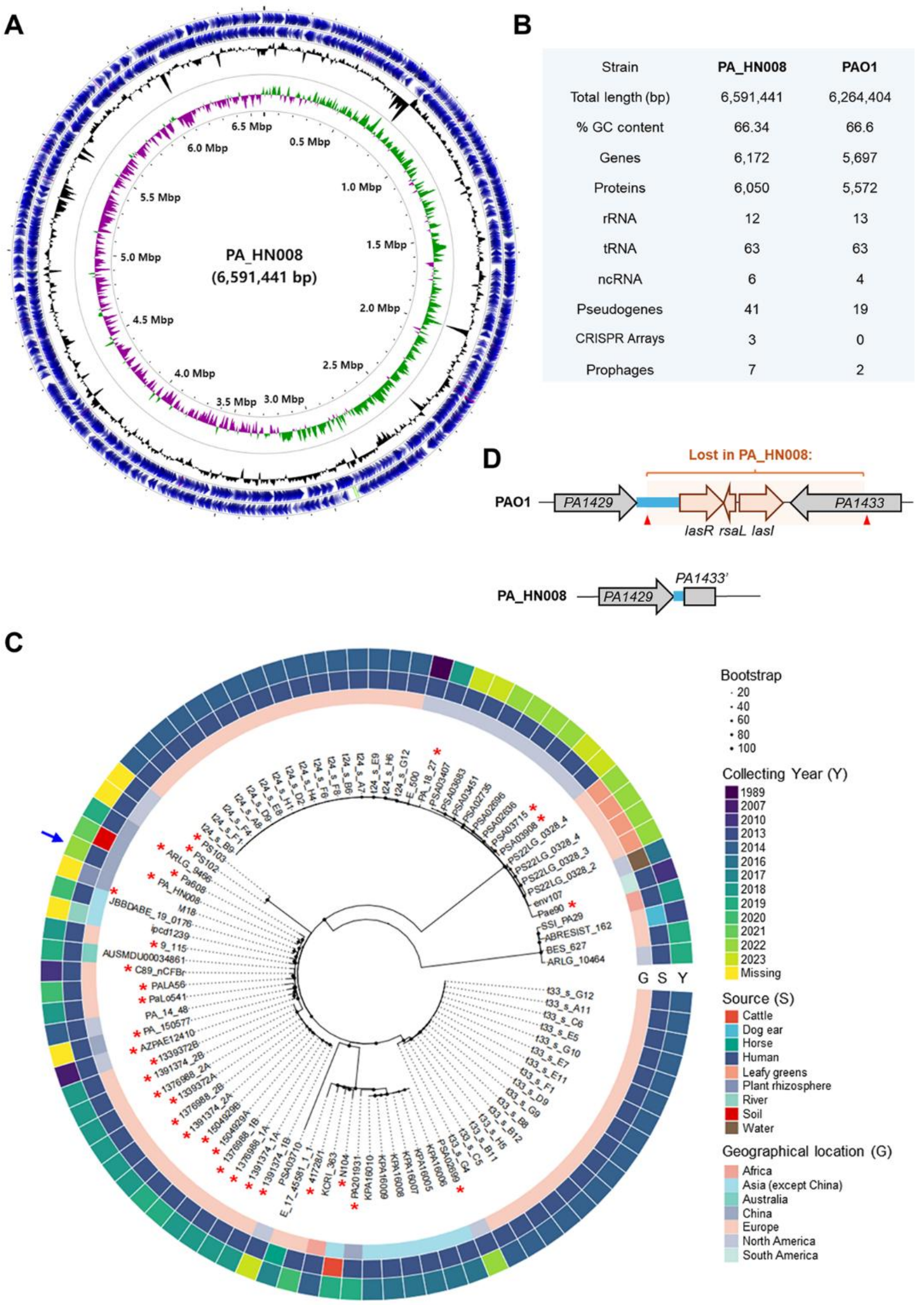

Figure S1. Genome information of PA_HN008 and phylogenetic distribution of *P. aeruginosa* strains belonging to the clonal complex of MLST1239. (**A**) Circular genome map of PA_HN008. (**B**) Genomic features of PAO1 and PA_HN008. (**C**) The phylogenetic tree was constructed based on 93 *P. aeruginosa* strains belonging to the same MLST1239 clonal complex. The blue arrow indicated the PA_HN008 strain. The information including the year (Y), source (S), and geographical location (G) of collecting is indicated with different colors of squares. Strain names accompanied by red stars indicated that the Las protein in this strain is not identical to that in PAO1. (**D**) A diagram showing the loss of the genetic fragment containing the *lasR*, *rsaL*, and *lasI* genes in PA_HN008.

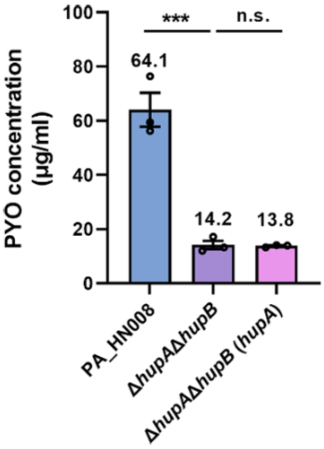

Figure S2. HupA regulates PYO production in a HupB-dependent manner. PYO production was quantified in PA_HN008 WT, Δ*hupA*Δ*hupB*, and Δ*hupA*Δ*hupB* (*hupA*) strains. Statistical significance was calculated based on one-way ANOVA (n.s., not significant; *P*< 0.001).

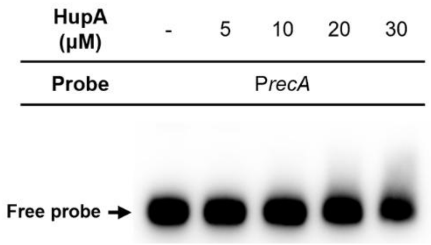

Figure S3. HupA does not bind to the promoter of *recA* (negative control).
